# In Vitro Comparative Skin Irritation Induced by Nano and Non-Nano Zinc Oxide

**DOI:** 10.3390/nano7030056

**Published:** 2017-03-04

**Authors:** Maria Pilar Vinardell, Hector Llanas, Laura Marics, Montserrat Mitjans

**Affiliations:** Department of Biochemistry and Physiology, Faculty of Pharmacy and Food Sciences, Universitat de Barcelona, Av. Joan XXIII 27-31, Barcelona 08028, Spain; hectorllanasmarco@gmail.com (H.L.); laura.marfa.89@gmail.com (L.M.); montsemitjans@ub.edu (M.M.)

**Keywords:** zinc oxide, skin irritation, cytotoxicity, keratinocytes, 3D-epidermal model

## Abstract

This study was designed to determine whether nano-sized ZnO has the potential to cause acute cutaneous irritation using cultured HaCaT keratinocytes and a human skin equivalent as in vitro models, compared to non-nanomaterials. Commercial nano ZnO with different sizes (50 nm and 100 nm) was characterized by dynamic light scattering (DLS) and microscopy (SEM) in different media. Nano ZnO reduced the cell viability of HaCaT in a dose-dependent and time-dependent manner, in a similar way to macro ZnO. However, the 3D-epidermis model revealed no irritation at 1 mg/mL after 24 h of exposure. In conclusion, nano-sized ZnO does not irritate skin, in a similar manner to non-nano ZnO.

## 1. Introduction

Nanotechnology is a powerful technology that has been used in a range of fields such as electronics and clothing, and in healthcare products as antimicrobial agents and drug delivery systems [1]. In particular, zinc oxide (ZnO) nanoparticles (NPs) are widely used in cosmetic and sunscreen products to protect against ultraviolet (UV)-induced skin damage, since they are transparent and scatter UV radiation [2]. However, there are many concerns about the potential toxicity of these new materials and nanotoxicology studies are therefore becoming increasingly common. As the field of nanotechnology has rapidly expanded, so too has the field of nanotoxicology [3].

Some studies have demonstrated the cytotoxic effect of ZnO nanoparticles on cancer cells and the efficacy of this effect against these cells has been studied [4]. This cytotoxic effect is one of the potential applications of these nanoparticles, but it is necessary to ensure they do not have a cytotoxic effect on normal cells.

Zinc oxide nanoparticles (ZnO NPs) exhibit novel physiochemical properties and their use in sunscreen and cosmetic products is increasing [5]. There is growing concern about their potential toxicity due to their close association with human skin [6]. It is well known that intact skin performs an important defensive role against a range of environmental stress factors. The skin acts as a barrier and provides a route of entry for foreign materials, including NPs such as zinc oxide nanoparticles. The high probability of contact between skin and NPs has raised concerns about the penetration of NPs and their subsequent interaction with skin cells [7]. There are many concerns related to the use of nanoparticles due to their higher surface area compared to the corresponding bulk form and their subsequent higher reactivity. This makes it necessary to study their potential for skin irritation compared to non-nano ZnO.

However, there is a lack of information about the negative health implications and toxicological impact of nanoscale ZnO particles, even though the processing and use of nanomaterials in everyday products is experiencing enormous growth. Major questions regarding the safety of ZnO NPs in sunscreens include their potential toxicity for viable human keratinocytes and their ability to penetrate the skin and any subsequent local and systemic distribution. Future studies should also concentrate on the relationship between particle size and skin penetration, and should consider specific NP surface properties in order to define detailed risk profiles for sunscreen ingredients.

Although ZnO NPs are widely used in cosmetics and sunscreen products, information on skin toxicity, including irritation, remains scarce [8]. Recent studies have shown no evidence of ZnO penetrating the superficial layers of the stratum corneum after application at a pH of 6 and 9 or within a sunscreen formulation [9]. Another study reported no adverse effects of ZnO (20 nm, negative charge) up to 1000 mg/kg body weight in either male or female rats after 90 days of dermal application [10]. The principal concern should therefore relate to their potential to cause skin irritation.

The European ban on animal-tested cosmetic products made it necessary to conduct in vitro studies of the safety of ZnO used in cosmetics. In this regard, studies that use keratinocytes as the principal skin cells or 3D-epidermal models represent a good strategy.

## 2. Results and Discussion

### 2.1. ZnO Characterization

The hydrodynamic size and polydispersity index of 50 nm and 100 nm ZnO in the different media studied are represented in Table 1.

Polydispersity index (PDI) values of around 0.3 were obtained in distilled water for 50 nm ZnO and for both nanoparticles in Dulbecco’s modified Eagle medium (DMEM). By contrast, significantly higher PDI values (1.0) were found for the NPs dispersed in phosphate buffered saline (PBS), which indicates a high dispersity in size.

Aggregation was observed in distilled water and especially in PBS due to the influence of the different salts present in the PBS [11]. By contrast, no aggregation was observed in DMEM in the case of 100 nm ZnO and low aggregation was observed for 50 nm ZnO. In DMEM, the presence of protein in the medium could form a protein corona around each particle that stabilizes the dispersion. Therefore, the real role of the protein corona should be investigated in further studies. The aggregation of different ZnO NPs is relevant to their basic properties and is influenced by electrolytes, which reduces their potential for penetration into cells to cause toxicity [12].

There were clear discrepancies in the size declared by the manufacturer, as revealed by the different methodologies used to determine the size of the nanoparticles. This is important information when comparing the different studies in the literature.

**Figure 1:**
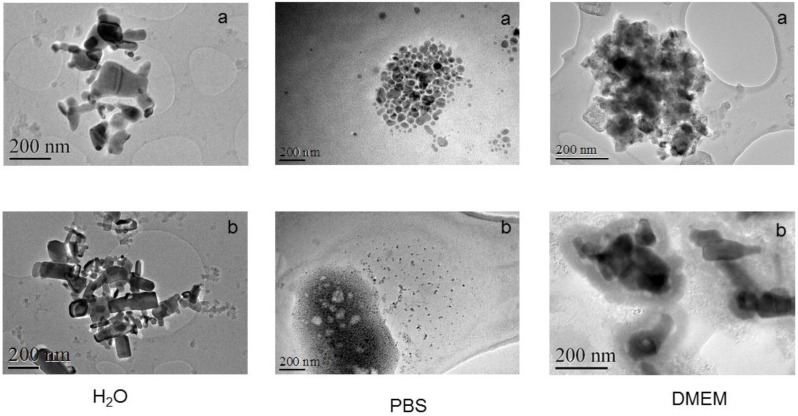
Transmission electron microscopy (TEM) images of ZnO 50 nm (**a**) and 100 nm (**b**) in distilled water, phosphate buffered saline (PBS) and Dulbecco’s modified Eagle medium (DMEM) cell culture medium, respectively.

The nanoparticles were characterized by transmission electron microscopy (TEM) in the different media, as shown in Figure 1.

The determined diameter of the particles was higher than that reported by the manufacturer due to aggregation in many cases. The higher diameter determined by ZnO of 50 nm compared to 100 nm should be attributed to a higher aggregation phenomena. The diameter determination is influenced by the procedure used in each case and that explains the discrepancies with the supplier information.

### 2.2. MTT Viability Assay in HaCaT Cells

No interference with the (3-(4,5-dimetylthiazol)-2,5 diphenyltetrazolium bromide (MTT) assay was observed at any concentration studied (data not shown), which demonstrated that the method was suitable for cytotoxicity determination.

**Figure 2:**
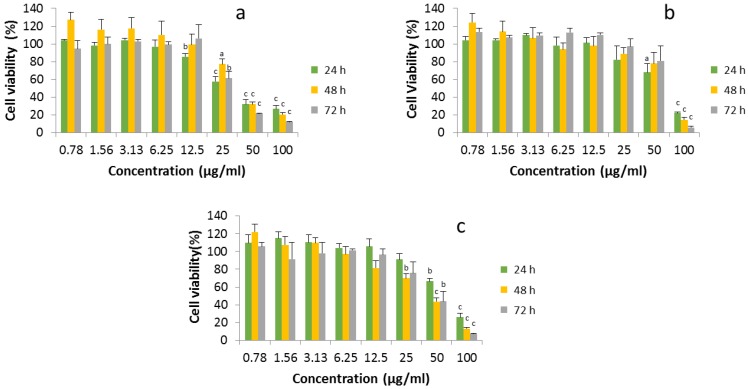
Cell viability of HaCaT cells exposed to different concentrations of ZnO (**a**) and ZnO 50 (**b**) and 100 nm (**c**), respectively, after 24, 48 and 72 h incubation. Mean values ± standard deviation of triplicates of at least three independent experiments. ^a^ *p* < 0.05, ^b^ *p* < 0.01 and ^c^ *p* < 0.001 compared to controls.

Figure 2 shows cell viability after the application of different concentrations (0.78 µg/mL to 100 µg/mL) of the ZnO studied for 24, 48 and 72 h. The three ZnO materials studied showed both a dose-dependent and time-dependent effect.

The viability of HaCaT cells treated with ZnO was also determined by other authors, but at a maximal time exposure of 24 h [13].

The cytotoxicity of ZnO nanoparticles to HepG2 cancer cells at the maximal concentration of 100 µg/mL is around five times higher than the cytotoxicity to HaCaT cells [14].

**Figure 3:**
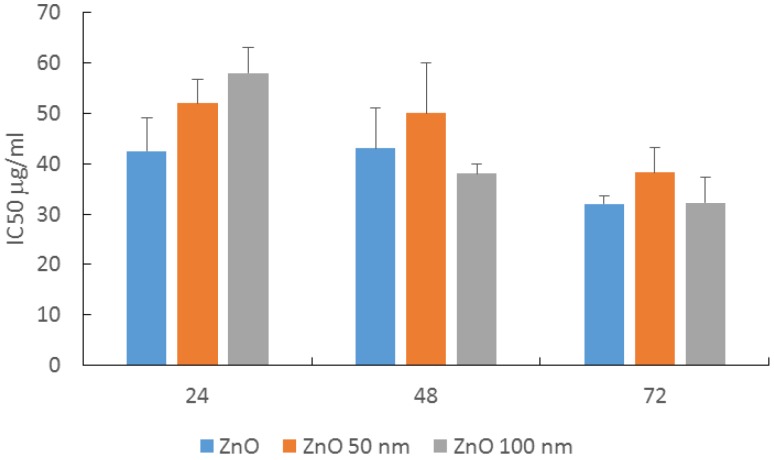
IC_50_ values calculated from the curves of cell viability of HaCaT cells exposed to ZnO and ZnO 50 and 100 nm after 24, 48 and 72 h incubation. Mean values ± standard deviation of triplicates of at least three independent experiments.

From the cytotoxicity results, we calculated the IC_50_ for the three ZnO materials at 24, 48 and 72 h, as shown in Figure 3.

At 24 h, there was a significant difference between the nano forms and the non-nanometric ZnO, with the non-nanometric ZnO the most cytotoxic and the 100 nm ZnO the least cytotoxic. The IC_50_ values decreased with the exposure time, but the cytotoxicity after 48 and 72 h of contact with cells was similar for the three ZnO materials studied, regardless of their size.

### 2.3. Effect on HaCaT Morphology

**Figure 4:**
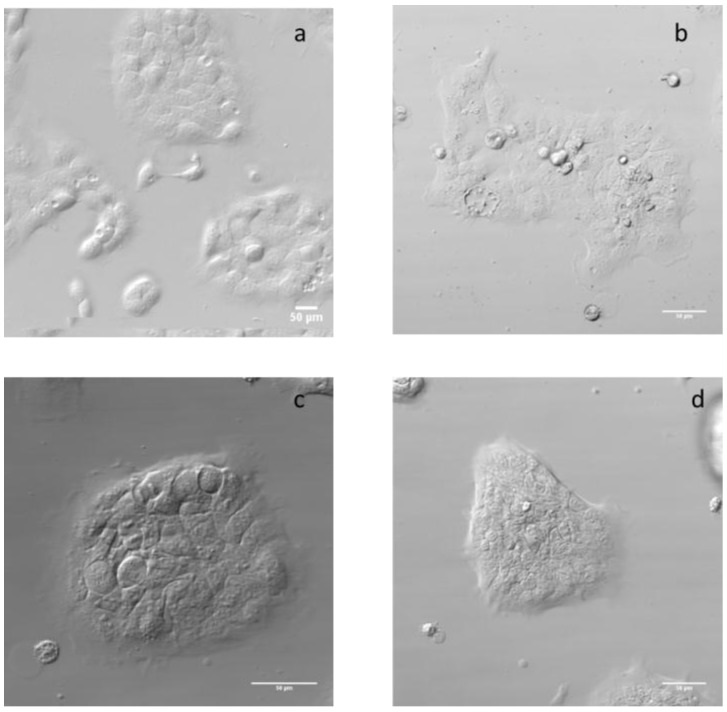
Images of HaCaT cell by phase contrast microscopy. Control cells without treatment (**a**), cells treated for 24 h with 25 µg/mL of ZnO (**b**), ZnO 50 nm (**c**) and ZnO 100 nm (**d**).

The HaCaT cells’ morphology before and after treatment was observed by phase contrast microscopy, which makes it possible to view unstained specimens, as shown in Figure 4. In line with our results, Lee et al. [13] also showed no effects on the morphology of HaCaT cells treated with 20 µg/mL of 22 nm ZnO after 24 h.

### 2.4. Skin Irritation on 3D Epidermis Model Study and Histology

We first applied the products for 15 min, as indicated by the Organization for Economic Co-operation and Development (OECD) skin irritation guidelines [15]. We did not observe any cytotoxic effects (data not shown). We then repeated the procedure, but this time we increased the contact time to 24 h to reflect a more realistic situation in which sunscreen is applied repeatedly. Results obtained with the Episkin model are presented in Table 2.

The percentage of viability of the treated models was around 100% for all ZnO materials, regardless of their size. The positive control, cells treated with 500 µg/mL of sodium dodecyl sulphate, showed a viability of around 21%, which demonstrated the irritant effect of this surfactant.

Similarly, no irritation was observed by other authors using smaller ZnO nanoparticles exposed to other 3D models for only 45 min [16], which is insufficient time to mimic the repeated application of sunscreen in a real situation.

Surekha et al., who studied 20 nm ZnO on rats, showed that, after repeated administration, low doses caused a reduction in collagen compared to a high dose and a control. However, these effects were reversible within a period of 14 days. From the above study, the authors concluded that nano ZnO may penetrate the skin at the above dosage levels and induce a reduction in collagen content [17].

Another study with 20 nm ZnO determined the induction of the proinflammatory cytokine TNF-α via an Egr-1-dependent mechanism in HaCaT cells. This induction seems to be the mechanism that regulates the ZnO-NP-induced inflammatory response [18]. However, this has not been studied with other ZnO nanoparticles and it is therefore difficult to draw any conclusions.

One of the main problems when interpreting results is the use of different ZnO nanoparticles with different sizes and origins, which makes it difficult to compare the results obtained in the different studies.

**Figure 5:**
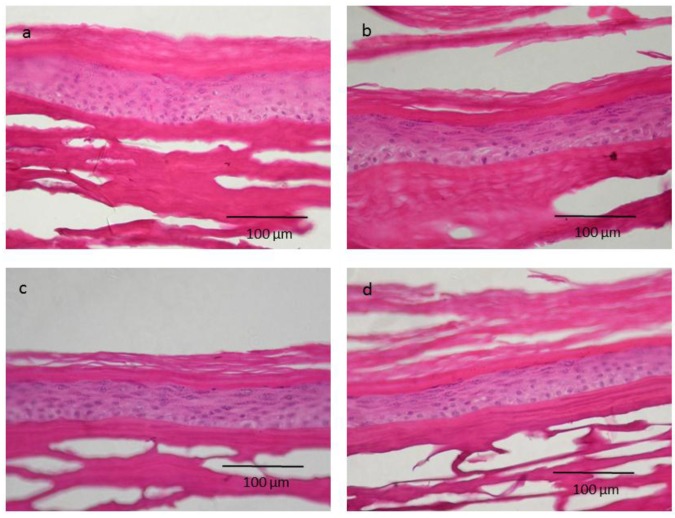
Episkin histological images. Tissues were processed for paraffin sections and stained with hematoxylin and eosin stain for histological examination. Normal control group treated with PBS (**a**), ZnO non-nanometric (**b**), ZnO 50 nm (**c**) and ZnO 100 nm (**d**).

As shown in Figure 5, human skin models treated with nanoparticle and non-nanoparticle ZnO showed a similar histology to the human skin model treated with PBS (control group).

A recent study showed that exposure of normal in vivo human skin to 75 nm ZnO nanoparticles under common in-use conditions of flexing or massage is not associated with significant adverse events [19]. This study corroborates our in vitro findings relating to the non-irritant effect of ZnO nanoparticles on skin and, thus, their safety.

In conclusion, the results obtained in this work suggest that there is no cause for concern in terms of the use of ZnO in nano form, since they show a similar or less pronounced effect on keratinocytes than the corresponding bulk form. When performing the studies on epidermal models that are considered more accurate for determining the potential for skin irritation of these chemicals, no irritant effects were observed after a large exposure such as a 24 h period.

## 3. Materials and Methods

### 3.1. ZnO Materials and Nanoparticles Characterization

ZnO in nano (50 nm and 100 nm) and micro forms were purchased from Sigma-Aldrich (St. Louis, MO, USA). Products are defined by manufacturer as ZnO nanopowder <50 nm particle size ZnO nanopowder, <100 nm particle size and ZnO ACS reagent 99%.

The nanoparticles were characterized by measuring their size in different media (distilled water, phosphate buffered saline (PBS) and DMEM cell culture medium with 5% FBS). The mean hydrodynamic diameter and polydispersity index (PDI) of the NPs were determined by dynamic light scattering (DLS) using a Malvern Zetasizer ZS (Malvern Instruments, Malvern, UK). The readings were taken at 25 °C, after 24 h incubation at 37 °C. Each measurement was performed using at least three sets of 10 runs.

The morphology and size of the NPs were analysed by transmission electron microscopy (TEM). A droplet (5 μL) of the NPs dispersed in distilled water was placed on a carbon-coated copper grid to form a thin liquid film.

### 3.2. Cell Culture and Treatment

HaCaT human keratinocytes (spontaneously immortalized human keratinocytes) were grown in DMEM medium (4.5 g/L glucose) supplemented with 10% (*v/v*) FBS, 2 mM l-glutamine, 100 U/mL penicillin and 100 µg/mL streptomycin at 37 °C, 5% CO_2_. These cells were routinely cultured in 75 cm^2^ culture flasks and trypsinized using trypsin-EDTA when the cells reached approximately 80% confluence. All cell lines were obtained from Eucellbank (Universitat de Barcelona, Barcelona, Spain).

To ensure that the dispersion was adequate, the ZnO-NP solutions were vortexed for 10 s prior to the test. The three ZnO materials were suspended in cell culture medium, and the cells were treated with 0.78 µg/mL to 100 µg/mL of ZnO for 24, 48 and 72 h. Each concentration was tested in triplicate and control cells were exposed to medium with 5% (*v/v*) foetal bovine serum only.

### 3.3. MTT Vibility Assay

Cytotoxicity was evaluated using thiazolyl blue tetrazolium bromide (3-(4,5-dimetylthiazol)-2,5 diphenyltetrazolium bromide (MTT), based on the reduction of the soluble yellow MTT triazolium salt to its blue insoluble MTT formazan product by mitochondrial succinic dehydrogenase as a measurement of cell metabolic activity [20]. Cell viability was calculated as the percentage of tetrazolium salt reduction by viable cells on each sample and the values were normalized by the untreated cell control (cells with medium only).

To study the potential interference of ZnO-NPs in the MTT assay as has been observed in some studies [21], the assay was also performed with only the ZnO-NPs without cells as was described previously [22].

### 3.4. Effect on HaCaT Morphology

The morphology of cells was determined after treatment with 25 μg/mL of the different ZnO materials for 24 h using phase-contrast microscopy.

### 3.5. Skin Irritation on 3D Epidermal Model and Histology

Episkin was purchased from Episkin (Lyon, France). It consists on a reconstructed human Epidermis kit of 0.38 cm^2^ and was used as a human skin equivalent model. and used as a human skin equivalent model. The EpiSkin models were transferred into the 12-well plates containing 1 mL of the pre-warmed assay medium and incubated for 24 h.

The medium was then replaced with pre-warmed fresh assay medium, 500 µg/mL of macro- and nano-sized ZnO diluted in PBS or PBS as the negative control or 500 µg/mL of SDS as the positive control were placed in cell culture inserts on top of the models for 15 min and 24 h. After the application time, any liquid remaining on top of the models was removed and the models were rinsed with PBS. The models were then placed into 12-well plates containing 300 µL of MTT solution and incubated for 3 h in a 5% CO_2_ incubator at 37 °C. They were then immersed in 500 µL of extraction solution (supplied with the models) for 2 h at room temperature. The absorbance of 200 µL of the extractant solution was measured in 96-well microtiter plates at 570 nm. The procedure was carried out in accordance with the manufacturer’s instructions.

### 3.6. Statistics

Mean ± standard deviation was calculated for triplicates for at least three independent experiments. Data were analyzed using software by one-way analysis of variance (ANOVA), followed by Tukey’s method. *p-*Values of < 0.05 were considered statistically significant.

## Figures and Tables

**Table 1:** Hydrodynamic size and polydispersity index (PDI) of ZnO of 50 and 100 nm determined in distilled water, phosphate buffered saline (PBS) and Dulbecco’s modified Eagle medium (DMEM) cell culture medium.

Nanoparticles	Distilled Water		PBS		DMEM	
	Hydrodynamic Size (nm)	PDI	Hydrodynamic Size (nm)	PDI	Hydrodynamic Size (nm)	PDI
ZnO 50 nm	208.7 ± 6.8	0.3 ± 0.0	969.8 ± 275.9	1.0 ± 0.0	239.8 ± 1.6	0.3 ± 0.0
ZnO 100 nm	1008.7 ± 329.0	0.4 ± 0.4	1120.7 ± 118.9	1.0 ± 0.1	93.1 ± 1.3	0.3 ± 0.0 ^1^

^1^ Mean value of at least three independent experiments ± SE.

**Table 2:** Viability of the Episkin model determined by MTT after treatment for 24 h with 500 µg/mL of different ZnO, 500 µg/mL of sodium dodecyl sulphate (SDS) as a positive control and PBS as a negative control. Mean value ± SD of triplicates.

% Viability	PBS	SDS	ZnO	ZnO 50 nm	ZnO 100 nm
Mean ± SD	100.00 ± 6.20	21.12 ± 5.52	109.84 ± 3.37	100.50 ± 14.27	102.77 ± 11.32
